# Do Alcohol Misuse, Service Utilisation, and Demographic Characteristics Differ between UK Veterans and Members of the General Public Attending an NHS General Hospital?

**DOI:** 10.3390/jcm5110095

**Published:** 2016-11-02

**Authors:** Dominic Murphy, Emily Palmer, Greta Westwood, Walter Busuttil, Neil Greenberg

**Affiliations:** 1 Combat Stress, Tyrwhitt House, Leatherhead KT22 0BX, UK; e.palmer@combat.org.uk (E.P.); w.busuttil@combat.org.uk (W.B.); 2 King’s Centre for Military Health Research, King’s College London, London SE5 9RP, UK; n.greenberg@combat.org.uk; 3 Portsmouth Hospitals NHS Trust, Portsmouth PO6 3LY, UK; G.westwood@combat.org.uk

**Keywords:** military, veterans, alcohol, substance misuse, NHS, mental health

## Abstract

The aim of this paper was to provide insights into alcohol misuse within UK veterans to inform as to whether their presentations differ from the general public. This was done by exploring differences in the severity of alcohol misuse between UK veterans and the general public admitted to a general NHS hospital over an 18 month period using retrospective data. All patients admitted to the hospital were screened for alcohol misuse. Those deemed as experiencing problems were referred for specialist nurse-led support. A total of 2331 individuals were referred for this supported and administered with a standardised assessment that included measures of the severity of alcohol difficulties (AUDIT), dependency levels (LDQ), and assessed for the presence of withdrawal symptoms (CIWA-Ar). In addition, information was collected on service utilisation, referral category (medical or mental health), other substance misuse, and demographic characteristics. No differences were found between the severity of reported alcohol difficulties between veterans and non-veterans. Evidence was found to suggest that veterans were more likely to be referred for support with alcohol difficulties at an older age and to be admitted to hospital for longer periods of time. This could have considerable cost implications for the NHS. It was more common for veterans to present at hospital with physical health difficulties prior to being referred for support for alcohol.

## 1. Introduction

Historically, military service has been associated with heavy alcohol consumption [1,2]. Furthermore, clear links have been demonstrated between alcohol consumption and negative impacts on well-being within military populations [3,4]. Alcohol has also been linked to violent offending post deployment [5]. Within the UK Armed Forces, prevalence rates of alcohol misuse of 13% have been reported [6]. The prevalence of alcohol related harm and alcohol dependence within the serving UK Armed Forces has been shown to be greater than in the UK general population [7]. Longitudinal studies of alcohol misuse in the UK military suggests that they are continuing to remain high [8]. These findings appear to be replicated in US samples [9,10]. The reasons for this disparity are unclear. Researchers have suggested several possible explanations; for instance, alcohol consumption may have been encouraged because of the belief that it fosters social cohesion when consumed in moderation, and alcohol tends to be readily available on military establishments and often at discounted prices [11]. In addition, military personnel are more at risk of being exposed to traumatic events and previous literature has observed a relationship between combat-exposure and alcohol misuse [7].

A pilot study commissioned by the UK Ministry of Defence found evidence to support the provision of veteran-specific clinics to support help-seeking and engagement with services for veterans with mental health difficulties [12]. Following this, the National Health Service (NHS) established veteran-specific local mental health services across the UK and commissioned Combat Stress to provide a national veteran-specific treatment programme for veterans with PTSD. Subsequently, evidence has emerged of greater numbers of UK veterans seeking support for mental health difficulties [13]. To date, veteran-specific substance misuse services have not been commission by the NHS.

The aim of this paper was to provide insights into alcohol misuse within UK veterans to inform as to whether their presentations markedly differ from the general public. This was done by exploring differences in terms of demographic characteristics, extent of alcohol and substance misuse, and service utilisation between veterans and members of the general public who were referred to an Alcohol Specialist Nurse Service (ASNS) following admission to an Acute Medical Unit (AMU) in a general hospital. ASNS teams are not a regular part of general hospital teams in the UK. They are specialist services that have been commissioned in a small number of hospitals across the UK that have identified high prevalence rates of problem drinkers amongst those being admitted. The role of the current ASNS team was to provide an alcohol misuse screening assessment for all patients admitted to the hospital. Patients who screened positive were then triaged for support by the ASNS team for either a medication detox with onward referral following their discharge to a community substance misuse service, onward referral only, or guided self-help.

## 2. Materials and Methods

### 2.1. Study Design and Sample

Retrospective data used for this study were extracted from the medical records of patients referred to the ASNS at a general district NHS Hospital in the south of England over an 18 month period between March 2014 and September 2015. During this period, all admissions to the AMU were screened for alcohol misuse using a Paddington Alcohol Test (PAT) [14]. The PAT had been modified for electronic usage. Those scoring six or above, indicating alcohol dependency, were referred to the ASNS for treatment and support. This resulted in 2331 individuals being referred to the ASNS. Data were collected on demographic characteristics, presenting admission diagnosis (medical or mental health), and alcohol and other substance misuse. Length of hospital stay was also recorded on discharge. An individual’s veteran status was not known to medical staff when they were first admitted to the hospital. This was only recorded during the ASNS assessment.

### 2.2. Measures of Alcohol Use

Three measures were used to assess alcohol use. These included the Alcohol Use Disorders Identification Test (AUDIT). The AUDIT has 10 items that explore consumption, dependence, and consequences of alcohol use over the previous 12 months. Scores can range from 0 to 40. Scores of between 8–15 indicate hazardous drinking, and scores of 16 or more highly harmful drinking [15]. The revised Alcohol Withdrawal Assessment (CIWA-Ar) was used to assess symptoms related to alcohol withdrawal. The CIWA-Ar includes 10 items. Nine of these items are scored between 0 and 7, and the remaining item between 0 and 4. Scores can range from 0 to 67. Scores of between 10 and 19 indicate mild to moderate withdrawal, and scores above 20 severe [16]. The Leeds Dependence Questionnaire (LDQ) was used to assess the psychological nature of alcohol dependence. The LDQ includes 10 items related to self-perceived alcohol dependence during the previous four week period. Items are scored 0 to 3. Scores can range from 0 to 30. Scores between 10 and 22 indicate medium dependence, and scores greater than 22 high levels of dependence [17].

### 2.3. Measures of Service Utilisation

Total length of hospital stay in days was recorded. Patients admitted and discharged in less than 24 h were coded as zero day stays. Many of the participants were admitted multiple times to the hospital over the 18 month study period. Two new variables were thus constructed; ‘total number of admissions’ and ‘total number of days stayed in hospital’ during this period.

### 2.4. Demographic Characteristics

Demographics data were collected and included age on admission, gender, employment status, and accommodation need (this referred to whether participants were experiencing a housing problem that needed support, for example, being at risk of eviction). A unique patient identification number was generated so that multiple admissions from the same individuals could be identified and whether other substance misuse was reported.

### 2.5. Statistical Analyses

Demographic characteristics, total number of admissions, and the number of unique participants for the sample were explored using descriptive statistics. Demographic characteristics were further assessed but this time stratified by participants who reported being a veteran and those who did not. This was done by fitting univariate logistic regression models to explore associations between demographics and veteran status. Multivariate logistic regression models were then fitted that included all the demographic variables. Where participants had been admitted multiple times during the study period, data for the above analyses was restricted to the first admission only.

The second stage of the analysis was to explore differences in service utilisation and alcohol use. Data from all admissions were used to do this. Clustering by unique identifier was used when performing the following analyses to take into account of within subject correlations. Comparison between veterans and non-veterans was made by calculating mean number of admissions, days per admission, length of stay, total number of days admitted to the hospital, and mean AUDIT, CIWA-Ar, and LDQ scores. Univariate linear regression was conducted between veteran status and each of these variables. The above regressions were refitted and adjusted for the demographic variables that had been found to significantly differ between veterans and non-veterans in the earlier analysis. Full details of the specific variables controlled for are given within the tables. All analyses were conducted using the statistical software package STATA version 13.0.

### 2.6. Ethical Approval

The study received ethical approval from the Combat Stress research ethics committee. This study was submitted for ethic review to the NHS. It was deemed to not require ethical approval because by the NHS because it used anonymised retrospective data.

## 3. Results

During the 18 month data collection period, 2331 patients admitted to the hospital were screened as experiencing alcohol difficulties and then referred to the ASNS. Thirty-one assessments did not take place because either the patient opted out of the referral or had been discharged prior to the planned assessment. Therefore, 2300 patients were seen and assessed by the ASNS. Of these 2300, 1663 were unique participants; many individuals had been referred for support more than once. The mean number of admissions for the whole sample was 1.38, and mean age 50.8 years old. One hundred and sixty-five of a total sample size of 1663 (9.9%) reported being a veteran.

Table 1 describes differences in the demographic characteristics between veterans and non-veterans. In the unadjusted analyses, veterans were significantly older (odds ratio (OR) 1.05 95% confidence interval (CI) 1.04 to 1.06) and retired (OR 3.49 95% CI 2.30 to 5.30) and significantly less likely to be female (OR 0.15 95% CI 0.09 to 0.27), unemployed (OR 0.60 95% CI 0.38 to 0.96), or using substances other than alcohol (OR 0.21 95% CI 0.19 to 0.46). When the analyses were adjusted, only being older, male and less likely to be unemployed remained significant. Thirty-four per cent of non-veterans initial presentations to the hospital were alcohol-related compared to 26% in the veteran group. However, this was not a significant difference. In the unadjusted analysis veterans were more likely to have been referred for alcohol support following an admission for a medical presenting difficulty than the non-veterans (OR 1.59 95% CI 1.03 to 2.45). However, this association became non-significant after adjustment for demographics, though it was approaching significance, which suggests we may have lacked sufficient power (OR 1.48 95% CI 0.97 to 2.33).

Service utilisation and alcohol use are reported in Table 2. No differences were observed between the mean number of hospital admissions for veterans and non-veterans. In the unadjusted analyses, there was evidence that each individual admission was longer for veterans (7.85 days versus 5.11 days); furthermore, over the 18 months, the total number of days that veterans had been admitted for was longer (11.6 days versus 7.0) than that of the non-veterans. After adjustment, these differences were no longer present.

No significant differences were observed on the severity of alcohol misuse measured by the AUDIT and CIWA-Ar between veterans and non-veterans. In a separate analysis, we explored AUDIT cut-off scores between the two groups. In veterans, 17% reported harmful drinking and 78% hazardous drinking, compared with 10% and 83% for non-veterans, respectively.

Dependency levels as measured by the LDQ appeared marginally higher for non-veterans when analyses were unadjusted (OR −2.55 95% CI −4.36 to −0.74). This difference lost significance following the addition of age, sex, and employment status as covariates.

Overall, for both veterans and non-veterans, mean scores on the AUDIT and LDQ were above recognised cut-offs, indicating high levels of alcohol problems and medium rates of dependency, respectively. Scores on the CIWA-Ar for both groups were just below the cut-off of eight, which indicated the need for prophylactic withdrawal medication.

## 4. Discussion

No differences were observed in the severity of alcohol difficulties between veterans and non-veterans. Veterans referred for support tended to be older, were more likely to be retired, and were more likely to be male. Given the general demographic make-up of military forces with high proportions of men (approx. 90% in the UK military), the latter of these is unsurprising [18]. Further, evidence has emerged suggesting that there are many barriers that prevent veterans from engaging in help-seeking behaviours [19,20,21]. The former differences suggest that veterans in this sample may have had a longer history of alcohol abuse that was not detrimental to their employment status. Before adjustment, our data suggested that veterans were admitted to hospital for longer periods of time. Whilst this seemed to be explained by adjusting for age, sex, and employment status, it does lead to the question of whether encouraging veterans to seek help sooner could reduce their service utilisation. Given the high costs associated with hospital admissions, a night stay in a general NHS ward has been estimated to cost approximately £350 [22], so finding ways to support veterans with alcohol misuse issues to seek help sooner appears prudent.

### 4.1. Strengths and Limitations

The major strengths of this study are that the sample was comprised of all individuals admitted to an AMU in a large general hospital over an 18 month period and who had been screened for alcohol misuse suggesting alcohol dependency. Given that there is evidence to suggest that only approximately 20% of veterans seek help for mental health and alcohol problems [20], this sampling strategy gave a unique insight into the alcohol-related needs of a sample of veterans and non-veterans who may not otherwise have come to the attention of traditional substance misuse services. Including all referrals over an 18 month period should improve the generalisability of our findings. However, several important limitations need to be considered. We were not able to access information about the initial PAT screenings for alcohol misuse that was administered to all individuals entering the hospital. This meant that we were unable to explore the differences in the proportions of veterans and non-veterans who were accessing the hospital who scored above and below the screening threshold on the PAT. This could have provided valuable information about whether veterans accessing the hospital were at increased risk of alcohol problems versus non-veterans as has been suggested in the literature [7]. Only demographic information collected during an individual’s first admission was used during analyses. Initial data checking suggested only modest changes in demographics occurred over the 18 month data collection period and that no differences in the patterns of these between veterans and non-veterans were observed. However, it is possible that other changes in variables that we were unable to collect occurred during the 18 month period, which could have affected veterans and non-veterans differently (for example, because of the different age profiles between the two groups). Information was not available on service characteristics of the veteran sample. For example, previous work within UK veterans has suggested an association between combat exposure and higher levels of problem drinking [7]. In addition, any inferences made within this paper could be restricted to a more general help-seeking population rather than those individuals explicitly seeking support for alcohol problems. This is because the sample appeared to be restricted to a mainly older population admitted to a general hospital. The hospital was a general hospital rather than one specialising in a particular area and so should not have introduced bias into the sample, but was located in an area within proximity of a large military base. As such, this could increase the likelihood of having a higher than average proportion of veterans residing within the catchment area.

### 4.2. Implications

The findings presented within the paper suggest that the alcohol difficulties of UK veterans do not differ significantly from the non-veterans in the general public. However, in this sample of those individuals accessing a district general hospital, veterans with alcohol problems appeared to be older and, before adjustment for age, more likely to be referred for medical difficulties rather than problems directly attributed to alcohol abuse. Taken together, this suggests that more support may be needed to help increase awareness in veterans about the potential harm of drinking higher than recommended levels and then help to engage veterans in alcohol services at a younger age. It could be helpful to use a case management system which encourages engagement with alcohol services to allow veterans earlier access to treatment. Our findings suggest that it is important for clinicians to work with veterans in a whole range of services, not limited to substance misuse problems, in order to be mindful of their clients’ alcohol intake.

Overall, our data suggest that only around 30% of the sample who were screened as having alcohol problems had actually accessed the hospital for treatment directly connected to alcohol difficulties. As such, this points to the efficacy of embedding specialist alcohol services within other clinical settings to support individuals who are experiencing difficulties but who might otherwise not be identified.

## 5. Conclusions

No differences in the severity of alcohol problems were observed between veterans and non-veterans who had been screened positive for experiencing difficulties with alcohol from a population attending a general hospital for a range of difficulties. However, veterans appeared to present over 10 years later, and there was some evidence to suggest they then spend longer periods of time admitted to hospital.

## Figures and Tables

**Table 1:** Demographic characteristics and presenting complaints between veterans and non veterans.

	Non-Veteran (%)	Veteran	Unadjusted Odds (95% CI)	Adjusted Odds ^a^ (95% CI)
*Age*				
Mean age	49.6	61.6	1.05 (1.04, 1.06) *	1.03 (1.01, 1.05) *
*Sex*				
Male	958/1498 (64)	152/165 (92)	1.00	1.00
Female	540/1498 (36)	13/165 (8)	0.15 (0.09, 0.27) *	0.15 (0.09, 0.28) *
*Employment status*				
Employed	409/1485 (28)	35/164 (21)	Reference group	1.00
Unemployed	778/1485 (52)	40/164 (24)	0.60 (0.38, 0.96) *	0.57 (0.35, 0.92) *
Retired	298/1485 (20)	89/164 (55)	3.49 (2.30, 5.30) *	1.68 (0.94, 3.01)
*Accommodation need*				
No problem	1278/1475 (87)	145/161 (90)	1.00	1.00
Housing problem	197/1475 (13)	16/161 (10)	0.72 (0.42, 1.22)	1.20 (0.67, 2.14)
*Substance problem*				
Alcohol only	1394/1498 (93)	163/165 (99)	1.00	1.00
Other substance as well	104/1498 (7)	2/165 (1)	0.21 (0.19, 0.46) *	0.37 (0.09, 1.55)
*Presenting complaint*				
Other	340/1467 (23%)	33/160 (21%)	1.00	1.00
Medical	486/1467 (33%)	75/160 (47%)	1.59 (1.03, 2.45) *	1.48 (0.97, 2.33)
Mental health	151/1467 (10%)	10/160 (6%)	0.68 (0.33, 1.42)	1.96 (0.89, 4.31)
Alcohol related	490/1467 (34%)	42/160 (26%)	0.88 (0.55, 1.42)	1.32 (0.79, 2.19)

^a^ Adjustment made for all other variables in table. * = *p* ≤ 0.05. Note. Numbers may not add up to 1663 because of missing data.

**Table 2:** Comparison of service utilisation and alcohol presentation between veterans and non-veterans.

	Non-Veteran	Veteran	Unadjusted β (95% CI)	Adjusted β ^a^ (95% CI)
*Number of admissions*				
Mean admissions	1.37	1.46	0.86 (−0.58, 0.23)	0.10 (−0.5, 0.25)
*Length of stay*				
Mean days	5.11	7.85	2.74 (1.06, 4.42) *	1.26 (−0.49, 3.01)
*Total number of days in hospital*				
Mean days	7.01	11.6	4.61 (1.82, 7.40) *	2.68 (-0.18, 5.54)
*AUDIT*				
Mean score	29.0	27.3	−1.66 (−3.35, 0.30)	−1.15 (−1.82, 1.51)
*CIWA-Ar*				
Mean score	7.58	7.68	0.10 (−1.21, 1.40)	1.14 (−0.19, 2.47)
*LDQ*				
Mean score	18.6	16.0	−2.55 (−4.36, -0.74) *	1.22 (−3.03, 0.58)

^a^ Adjustment age, sex, and employment status. * = *p* ≤ 0.05.
